# Epigenetic Regulation of TET1-SP1 During Spermatogonia Self-Renewal and Proliferation

**DOI:** 10.3389/fphys.2022.843825

**Published:** 2022-02-11

**Authors:** Lingling Liu, Jin Wang, Shenghua Wang, Mudi Wang, Yuanhua Chen, Liming Zheng

**Affiliations:** School of Basic Medical Sciences, Anhui Medical University, Hefei, China

**Keywords:** TET1, SP1, epigenetic modification, self-renewal, spermatogonia

## Abstract

Spermatogonia are the source of spermatogenic waves. Abnormal spermatogonia can cause ab-normal spermatogenic waves, which manifest as spermatogenic disorders such as oligospermia, hypospermia, and azoospermia. Among them, the self-renewal of spermatogonia serves as the basis for maintaining the process of spermatogenesis, and the closely regulated balance between self-renewal and differentiation of spermatogonia can maintain the continuous production of spermatozoa. Tet methylcytosine dioxygenase 1(TET1) is an important epitope modifying enzyme that catalyzes the conversion of 5-methylcytosine (5-mC) to 5-hydroxymethylcytosine (5-hmC), thereby causing the methylation of specific genes site hydroxylation, enabling the DNA de-methylation process, and regulating gene expression. However, the hydroxymethylation sites at which TET1 acts specifically and the mechanisms of interaction affecting key differential genes are not clear. In the present study, we provide evidence that the expression of PLZF, a marker gene for spermatogonia self-renewal, was significantly elevated in the TET1 overexpression group, while the expression of PCNA, a proliferation-related marker gene, was also elevated at the mRNA level. Significant differential expression of SP1 was found by sequencing. SP1 expression was increased at both mRNA level and protein level after TET1 overexpression, while differential gene DAXX expression was downregulated at protein level, while the expression of its reciprocal protein P53 was upregulated. In conclusion, our results suggest that TET1 overexpression causes changes in the expression of SP1, DAXX and other genes, and that there is a certain antagonistic effect between SP1 and DAXX, which eventually reaches a dynamic balance to maintain the self-renewal state of spermatogonia for sustained sperm production. These findings may contribute to the understanding of male reproductive system disorders.

## Introduction

According to research estimates, approximately 8–12% of couples worldwide are deeply affected by infertility, with approximately 50% of infertility being due to problems with the male partner (Minhas et al., 2021). The presence of male infertility may be associated with impaired sperm production due to congenital, genetic, or idiopathic factors (Vander Borght and Wyns, 2018). There is relevant evidence that when a mother conceives, the health status of the father at this time can influence the level of reproductive health of the offspring through the inheritance of epigenetic modifications (Craig et al., 2017). Epigenetic modifications play an important role in germ cell function and post-fertilization embryonic development. In order to form terminally differentiated spermatozoa and promote the totipotency of fertilized eggs, these epigenetic modifications must be precisely regulated. Male infertility or early embryonic dysplasia may be associated with reproductive disorders resulting from epigenetic alterations associated with the male reproductive process (Liu et al., 2019). Epigenetic modifications can be involved in the spermatogenesis process and affect the fate of spermatogonia by regulating reproduction-specific genes. Self-renewal is fundamental to maintaining the spermatogenesis process, and abnormal self-renewal of spermato-gonia leads to reduced stability, causing decreased fertility, which eventually manifests as testicular atrophy or even infertility (Zheng et al., 2016).

The DNA hydroxymethylase TET1 is an important epitope-modifying enzyme (Ross and Bogdanovic, 2019). The TET1 protein possesses a catalytic structural domain with α-ketoglutarate (α-KG) and Fe2+ binding sites near the carbon terminus, a cyste-ine-rich region in front of the catalytic structural domain, and a CXXC structural domain with recognition function near the nitrogen terminus (Rasmussen and Helin, 2016), which can be directly recognized and bound to DNA to facilitate recruitment of genomic targets (Zhang et al., 2010). TET1 can catalyze the conversion of 5-methylcytosine (5-mC) to 5-hydroxymethylcytosine (5-hmC), which can further be converted to 5-formylcytosine (5fC) and 5 carboxycytosine (5caC). It is then recognized and excised by thymine—DNA glycosylase (TDG) and subsequently converted to cyto-sine *via* the base excision repair pathway (BER), which hydroxylates the methylation sites of specific genes, thus enabling the process of DNA demethylation and regulation of gene expression (Lio et al., 2019). Also, the hydroxylation product 5hmC can regulate gene transcription through its own recruitment (Cui et al., 2020). Localization distribution analysis at the genome-wide level revealed that TET1 and its hydroxylation product 5hmC are mainly distributed in many promoters, exons, transcription initiation regions and other important locations, which also suggests that TET1-mediated demethylation is closely linked to gene transcriptional activity (Wu and Morris, 2001; Wu et al., 2011).

Sp1 transcription factor (SP1) is a known member of the transcription factor family that also includes SP2, SP3, and SP4, which are involved in a variety of important biological processes (Peng et al., 2020). The structure of SP1 possesses three highly homologous C2H2 regions that feature direct binding to DNA and therefore enhance the transcriptional activity of genes (Memon and Lee, 2018). The SP family has a highly conserved DNA-binding structural domain (C-terminal structural domain), while the N-terminal region varies, so it is through this structural domain that many transcription factors regulate gene transcription (Zhang et al., 2013). The proteins encoded by SP1 can be involved in many cellular processes, such as cell growth, cell differentiation, apoptosis, immune response, chromatin remodeling, and DNA damage (Xu et al., 2019). Interestingly, SP1 not only initiates transcription but also has a regulatory role in activating or repressing processes (Vellingiri et al., 2020). It has been found that SP1 can activate gene transcription in many cells, and the promoter regions of these activated genes contain abundant GC binding sites (Vizcaíno et al., 2015). SP1 target genes are mainly involved in cell proliferation as well as tumorigenesis (Guo et al., 2019). Previous studies have found that the SP family is commonly over-expressed in certain human cancers and therefore is often considered as a negative prognostic factor (Beishline and Azizkhan-Clifford, 2015). When SP1 is overexpressed, it can contribute to the malignant phenotype of various human cancers by upregulating a number of genes associated with proliferation, invasion and metastasis, as well as certain genes with stem-ness and chemoresistance, resulting in a negative prognosis (Dupuis-Maurin et al., 2015).

The biological functions of DAXX (death domain associated protein) are complex. Previous studies have found a noteworthy commonality among various cancers in that DAXX is overexpressed in a variety of cancers and its possible association with tumorigenesis, disease progression and treatment resistance (Mahmud and Liao, 2019). DAXX was identified in 1997 as a regulator of FAS-binding protein and Jun N-terminal kinase (JNK)-mediated cell death (Yang et al., 1997; Chang et al., 1998). DAXX is almost ubiquitous in human tissues and its role in embryonic development is also crucial (Bogolyubova and Bogolyubov, 2021). DAXX can bind to a variety of DNA through transcription factors (TFs), chromatin-associated proteins, core histones, epigenetic regulators, etc., to regulate gene expression as transcriptional co-repressors or co-activators (Ivanauskiene et al., 2014; Nye et al., 2018; Wasylishen et al., 2018; Heaphy et al., 2020).

Previous studies have found that TET1 can participate in the spermatogenesis process and affect the self-renewal and proliferation of spermatogonial stem cells (SSCs; Zheng et al., 2016), but the hydroxymethylation sites of TET1 specific action and the mechanism of interactions affecting key differential genes are not clear. Therefore, in this study, by over-expressing TET1 in spermatogonia and discovering the differential methylation sites of TET1 hydroxylation by sequencing, and combining with mRNA level and protein level analysis, we further explain the epigenetic regulation mechanism of TET1 on spermatogonia self-renewal from the epigenetic level, which provides scientific basis for studying spermatogenesis, revealing the causes leading to spermatogenic disorders, and elucidating the mechanism of this will provide important scientific clues for the study of spermatogenesis, reveal the causes of spermatogenesis disorder, and elucidate its mechanism, and provide important scientific clues for cytogenetic treatment of male infertility.

## Materials and Methods

### Cell Culture and Plasmid Transfection

Mouse spermatogonia GC-1 cells were used for cell culture, complete medium was made by adding 10% fetal bovine serum (FBS) to the basal medium DMEM of Hyclone, and cells were cultured in a humidified environment containing 5% carbon dioxide at 37°C. Passage was performed every 3 days. At the time of passage, digestion was stopped by adding complete medium containing serum after digestion with trypsin digestion solution (Beyotime) containing .25% trypsin and .02% ethylene diamine tetraacetic acid (EDTA) for 2 min. When the Mouse spermatogonia GC-1 cells were cultured with the density of 75%, fresh DMEM consisted of FBS and other supplements were replaced. 30 min later, Lipofectamine 3000 reagent (Thermo) and plasmid (MYC and MYC-TET1) were, respectively, co-incubated with a volume V(MYC) = (2,500 ng/600 ng/ul) and V(MYC-TET1) = (2,500 ng/800 ng/ul) at room temperature for 15 min, then added to the medium and blended them. 12 h later, fresh DMEM contained all the supplements were replaced.

### After 24–48 h Transfection, the Transfected Cells Were Collected for Subsequent Analysis. Real-Time Reverse Transcriptase Polymerase Chain Reaction

Twenty-four hours after cell transfection, transfected cells were collected, with Trizol (Thermo) Total RNA was purified and purified with cDNA was synthesized by a reverse transcription kit (SPARKscript II RT Plus Kit), followed by the use of SYBR Green qPCR Mix for SPARK and a fluorescence quantitative PCR instrument (ABI, QuantStudio6 Flex) Real-time reverse transcription-polymerase chain reaction (RT-PCR) was performed. GAPDH as a housekeeping gene for the normalization of gene expression. Primers were synthesized by Shanghai Shenggong. Primer pairs used in the experiments are listed in following Table 1.

**Table 1:** QRT-PCR primers.

Gene	Forward	Reverse
GAPDH	TGGCCTTCCGTGTTCCTAC	GAGTTGCTGTTGAAGTCGCA
TET1	GAGCCTGTTCCTCGATGTGG	CAAACCCACCTGAGGCTGTT
PLZF	CACCGCAACAGCCAGCACTAT	CAGCGTACAGCAGGTCATCCAG
GFRα1	GACCGTCTGGACTGTGTGAAAG	TTAGTGTGCGGTACTTGGTGC
PCNA	AGTGGAGAACTTGGAAATGGAA	GAGACAGTGGAGTGGCTTTTGT
Cylin A	TGGCTGTGAACTACATTGA	ACAAACTCTGCTACTTCTGG
Cylin E	GTGGCTCCGACCTTTCAGTC	CACAGTCTTGTCAATCTTGGCA
MAGE4	ATGGAAAATCCCGATAACACCC	AGGACTTGGTAATCCACTACTGT
PRDM1	TGGAGGACGCTGATATGACT	CTTACCACGCCAATAACCTC
VASA	GATAATCATTTAGCACAGCCTC	GTCAACAGATGCAAACACAG
DAZL	ATGTCTGCCACAACTTCTGAG	CTGATTTCGGTTTCATCCATCCT
C-KIT	CGCCTGCCGAAATGTATG	TCAGCGTCCCAGCAAGTC
SP1	GGCAGCGAGTCTTCCAAGAA	GATGATCTGTTGGTTTGCACCT

### Western Blot

After 48 h of cell transfection, transfected cells were collected. Proteins were extracted from transfected cells, and protein concentrations were determined with a BCA protein quantification kit (P0010S, Beyotime). Protein samples were denatured by 5% SDS-PAGE sample loading buffer, solubilized with 10% SDS-PAGE, and transferred to PVDF membranes. Detection was performed with β-actin (.5 ug/ml, GenScript#A00702S#Mouse) and anti-SP1 (1:1,000; Boster#A00110-1#Rabbit), anti-PLZF (1:1,000; Boster#PB1010#Rabbit), anti-GFRα1 (1:1,000; Boster#PB0199#Rabbit), anti-P53 (1:1,000; Bioworld#BS6437#Rabbit) and anti-DAXX (1:1,000; Bioworld#BS2411#Rabbit). Horseradish peroxidase-conjugated anti-rabbit (1:5,000, Boster#BA1054) and anti-mouse (1:10,000, Boster#BA1050) were used as secondary antibodies. The substrates were detected with a high-sensitivity ECL chemiluminescence kit (P0018S, Beyotime), and the results were analyzed with a Tanon-5200 automated gel imaging system.

### Immunofluorescence Microscopy

Immunofluorescence staining of cells: cells cultured *in vitro* were washed twice with phosphate buffered saline (PBS), fixed with 4% paraformaldehyde (PFA) for 15 min at room temperature, and washed twice with PBS for 5 min each time. The membrane was permeabilized with .1% TritonX-100 for 10 min at room temperature and then washed twice with PBS for 5 min. If the protein is a membrane protein, omit this step. After washing with PBS three times for 5 min each, they were blocked with 1% bovine serum albumin (BSA) for 1 h at room temperature and then incubated with SP1 primary antibody (1:500, Boster#A00110-1#Rabbit) overnight at 4°C. After three washes with PBS, they were incubated with the appropriate secondary antibody (DyLight 488, Goat Anti-rabbit IgG, Boster# BA1127) for 40 min at room temperature. After the slides were washed with PBS, 1 μg/ml of nucleic acid fuel DAPI (biosharp#BS097) was added. Images were captured using a Nikon inverted fluorescence microscope.

### Transcriptome Sequencing and Protein Sequencing Data

The data discussed in this publication have been deposited in NCBI’s Gene Expression Omnibus and are accessible through GEO Series accession number GSE193717.^1^

The mass spectrometry proteomics data have been deposited to the ProteomeXchange Consortium *via* the PRIDE partner repository with the dataset identifier PXD030967.

### Statistical Analysis

Data were expressed as means ± SD (*n* ≥ 3) and analyzed using GraphPad Prism 7 (GraphPad Software, San Diego, CA). Firstly, the data were verified to conform to normal distribution and homogeneity of variance. Comparison between two groups was analyzed using the *t*-test. Comparison among groups was analyzed using one-way ANOVA. If the data did not conform to normal distribution or homogeneity of variance, the rank-sum test was employed for the nonparametric analysis. Statistical significance was defined as *p* < .001(***), *p* < .01(**), or *p* < .05(*).

## Results

### TET1 Overexpression Maintains Self-Renewal and Accelerates Proliferation of Spermatogonia

**Figure 1:**
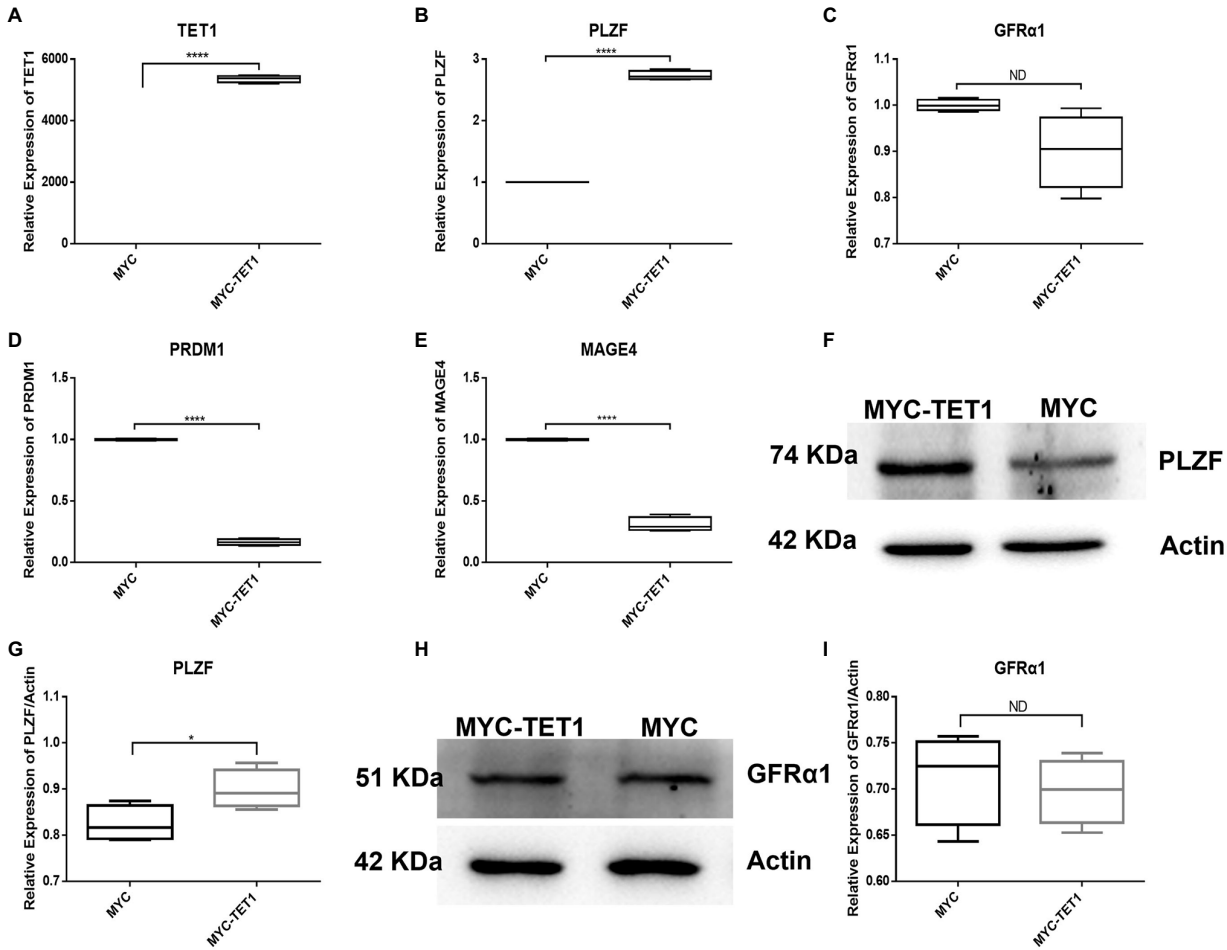
Relative expression of specific genes in spermatogonial cells. **(A)** The mRNA expression of TET1 in spermatogonial cells was detected by QRT-PCR. **(B)** The mRNA expression of PLZF in spermatogonial cells was detected by QRT-PCR. **(C)** The mRNA expression of GFRα1 in spermatogonial cells was detected by QRT-PCR. **(D)** The mRNA expression of PRDM1 in spermatogonial cells was detected by QRT-PCR. **(E)** The mRNA expression of MAGE4 in spermatogonial cells was detected by QRT-PCR. **(F)** The expression of PLZF in TET1 overexpressed cells was detected by Western Blot. **(G)** Quantification of PLZF protein levels in TET1 overexpressed cells. **(H)** The expression of GFRα1 in TET1 overexpressed cells was detected by Western Blot. **(I)** Quantification of GFRα1 protein levels in TET1 overexpressed cells. *p* < .0001(****), *p* < .001(***), *p* < .01(**), *p* < .05(*).

To identify the effect of TET1 overexpression on spermatogonia self-renewal and proliferation, we examined spermatogonia-specific related genes in the TET1 overexpression group and control cells, respectively. QRT-PCR results showed that the mRNA expression level of TET1 was significantly increased in TET1 overexpression cells (Figure 1A), and PLZF, which is related to spermatogonia self-renewal, had its mRNA level expression increased significantly (Figure 1B) and its protein level expression also increased to some extent (Figures 1F,G), the changes of mRNA expression level and protein level of GFRα1 were not obvious (Figures 1C,H,I), and the mRNA expression level of MAGE4 and PRDM1 decreased (Figures 1D,E), the above results indicate that TET1 has maintained the function of spermatogonia self-renewal.

**Figure 2:**
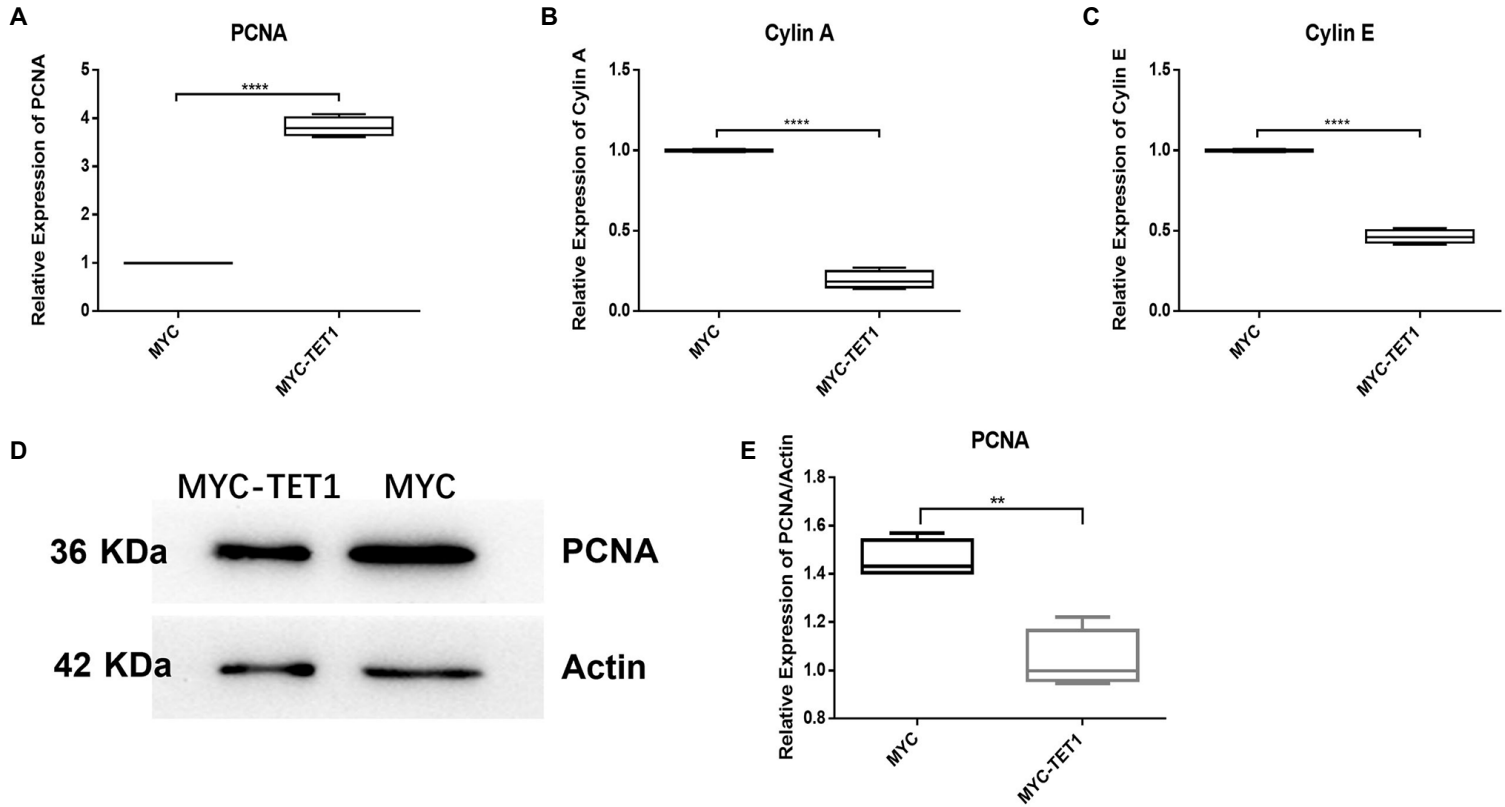
Relative expression of specific genes in spermatogonial cells. **(A)** The mRNA expression of PCNA in spermatogonial cells was detected by QRT-PCR. **(B)** The mRNA expression of Cylin A in spermatogonial cells was detected by QRT-PCR. **(C)** The mRNA expression of Cylin E in spermatogonial cells was detected by QRT-PCR. **(D)** The expression of PCNA in TET1 overexpressed cells was detected by Western Blot. **(E)** Quantification of PCNA protein levels in TET1 overexpressed cells. *p* < .0001(****), *p* < .001(***), *p* < .01(**), *p* < .05(*).

**Figure 3:**
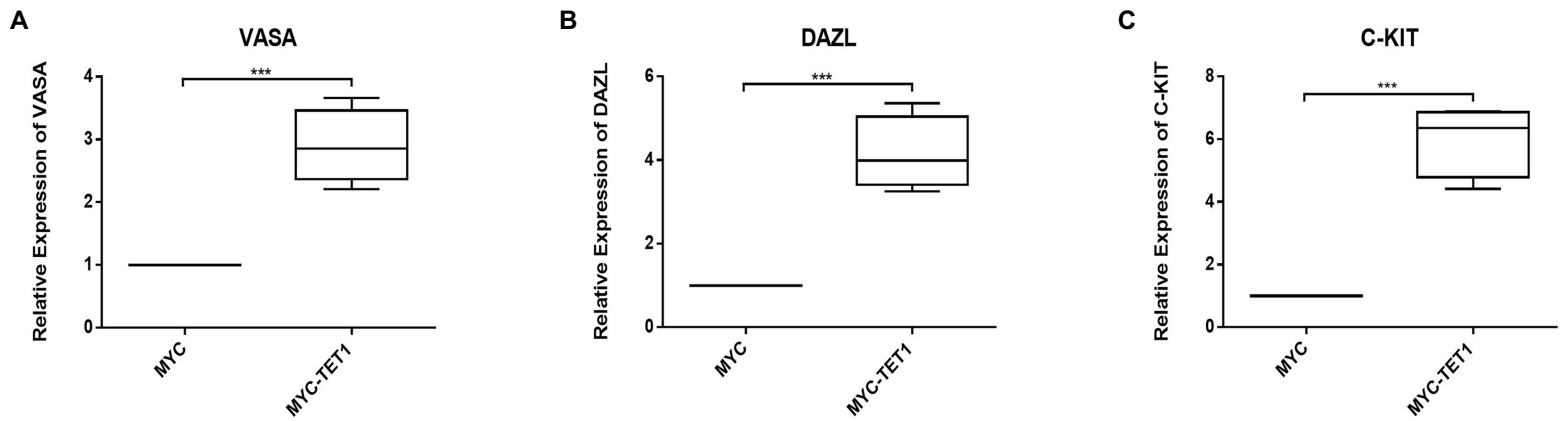
Relative expression of specific genes in spermatogonial cells. **(A)** The mRNA expression of VASA in spermatogonial cells was detected by QRT-PCR. **(B)** The mRNA expression of DAZL in spermatogonial cells was detected by QRT-PCR. **(C)** The mRNA expression of C-KIT in spermatogonial cells was detected by QRT-PCR. *p* < .001(***), *p* < .01(**), *p* < .05(*).

Although the mRNA expression levels of Cylin A and Cylin E, which are related to the cell cycle, were somewhat decreased in TET1 overexpression cells (Figures 2B,C), the expression of PCNA, a gene specific for cell proliferation, was significantly increased at the mRNA level (Figure 2A). At the protein level, the expression level of PCNA decreased (Figures 2D,E), indicating that TET1 overexpression enhanced cell transcription and favored cell proliferation. The mRNA expression levels of VASA, DAZL, and C-KIT, which are associated with spermatogonial differentiation, were all increased to some extent (Figures 3A–C), indicating that TET1 can promote spermatogonial differentiation. All the above results indicate that TET1 overexpression can maintain the self-renewal state of spermatogonia, which leads to enhanced cell transcription and facilitates cell proliferation and differentiation.

### Analysis of Global mRNA Levels After TET1 Overexpression

**Figure 4:**
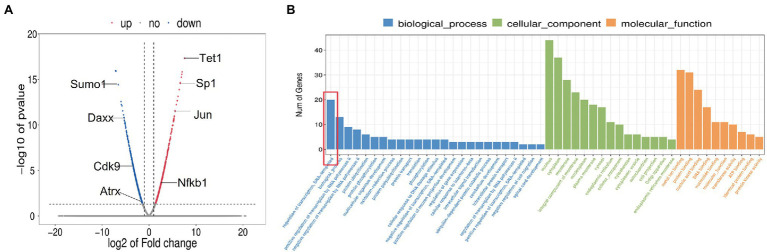
Sequencing results of 36 h transcriptome of TET1 overexpression group and control group. **(A)** Transcriptome 36 h differential gene volcano map. The abscissa represents the differential expression multiple changes of genes in different samples. The ordinate represents the statistical significance of changes in gene expression levels. Red represents significantly upregulated differentially expressed genes, blue represents significantly downregulated differentially expressed genes, and gray dots represent nonsignificantly differentially expressed genes. **(B)** Histogram of GO enrichment of differential genes.

To identify the dynamic changes in the overall mRNA levels in spermatogonia after TET1 gene overexpression, we examined TET1 overexpression cells and control cells by RNA-seq and QRT-PCR techniques. We used differential multiplicity FC > =2 or FC < = .5 (that is, the absolute value of log2FC > = 1) and value of *p*<.05 as the criteria, and the genes thus screened were differentially expressed genes (DEGs). The sequencing results showed that there were 455 differentially expressed genes, of which 195 genes were upregulated at the mRNA level and 260 genes were downregulated at the mRNA level (Figure 4A). Using GO analysis, we found that genes involved in transcriptional regulation were significantly enriched in TET1 overexpressing spermatogonia (Figure 4B). This result suggests that TET1 overexpressing cells are more conducive to cellular transcription than control cells, resulting in enhanced cellular activity.

### SP1 and DAXX Co-regulate Self-Renewal of Spermatogonia

**Figure 5:**
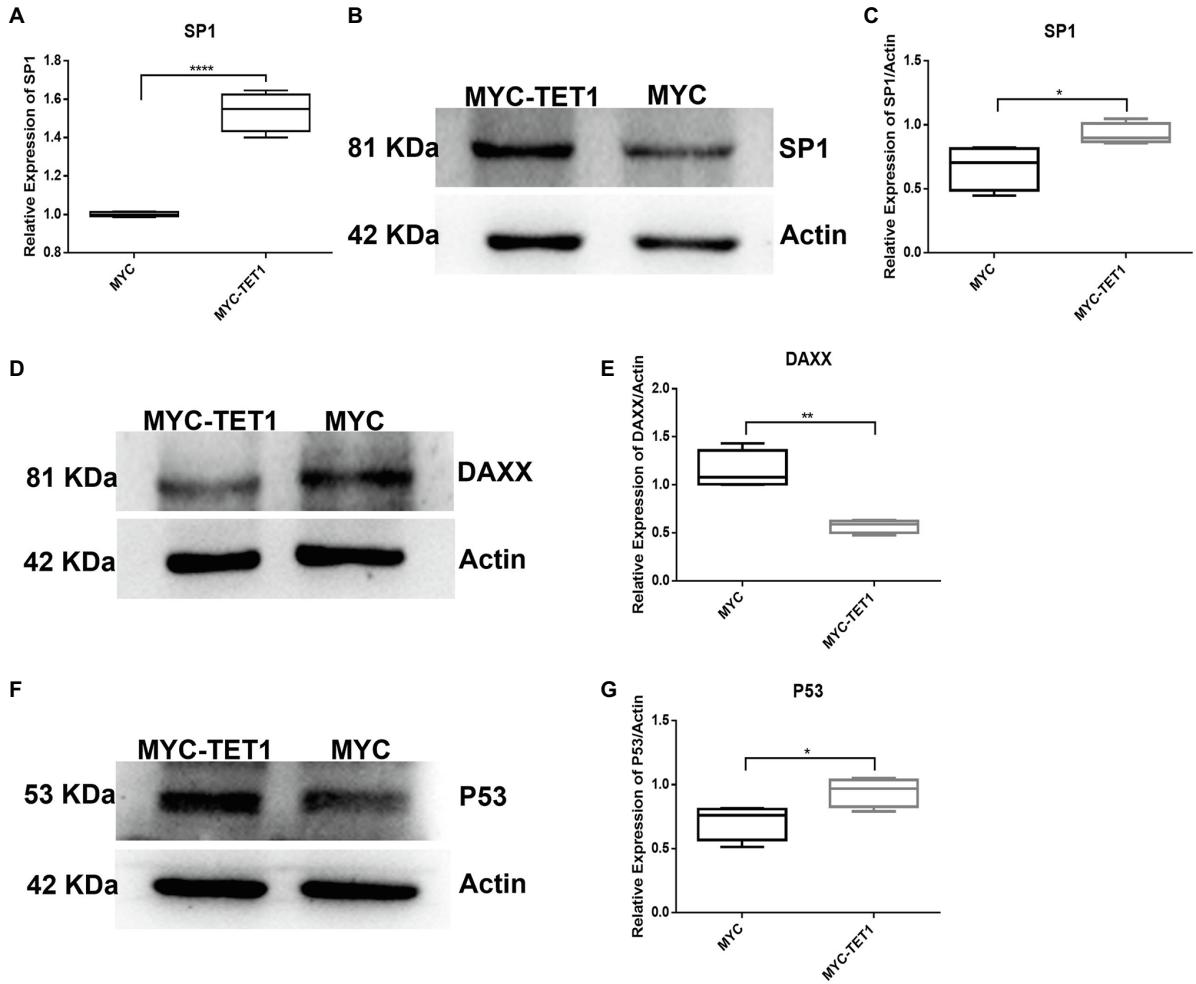
mRNA and protein levels of TET1 and MYC-TET1 cells were detected. **(A)** The mRNA expression of SP1 in spermatogonial cells was detected by QRT-PCR. **(B)** The expression of SP1 in TET1 overexpressed cells was detected by Western Blot. **(C)** Quantification of SP1 protein levels in TET1 overexpressed cells. **(D)** The expression of DAXX in TET1 overexpressed cells was detected by Western Blot. **(E)** Quantification of DAXX protein levels in TET1 overexpressed cells. **(F)** The expression of P53 in TET1 overexpressed cells was detected by Western Blot. **(G)** Quantification of P53 protein levels in TET1 overexpressed cells. *p* < .0001(****), *p* < .001(***), *p* < .01(**), *p* < .05(*).

**Figure 6:**
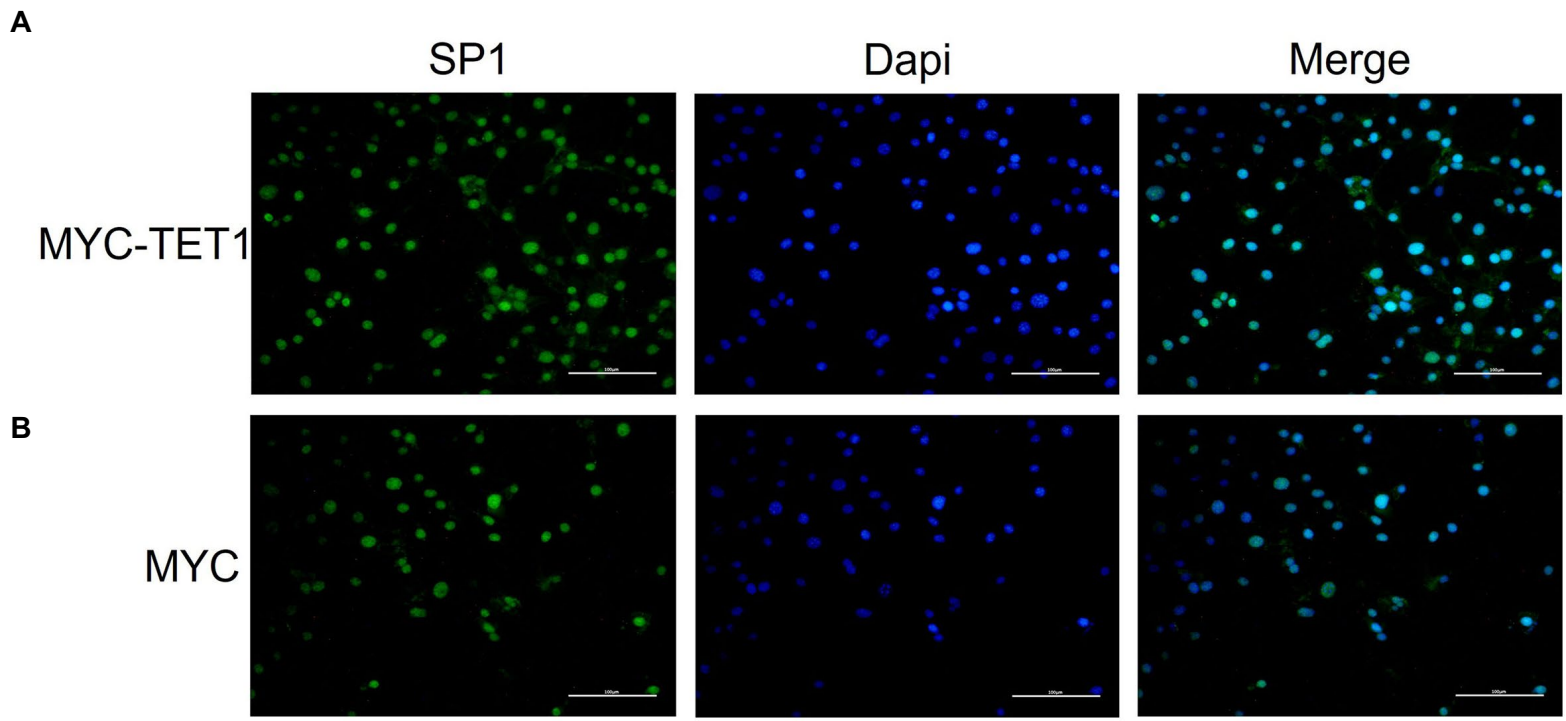
Immunofluorescence staining of SP1 in overexpressed and control cells cultured *in vitro*. **(A)** Immunofluorescence staining of SP1 in overexpressed cells (MYC-TET1) cultured *in vitro*. **(B)** Immunofluorescence staining of SP1 in control cells (MYC) cultured *in vitro*.

After determining the fluctuating changes in overall mRNA levels after TET1 overexpression, we analyzed the 195 upregulated differential genes and found that SP1 was significantly different (Figure 4A). QRT-PCR results showed that the mRNA level expression of SP1 increased after TET1 overexpression (Figure 5A), and WB detection revealed that SP1 was also expressed at the protein level (Figures 5B,C), and then we performed immunofluorescence staining of the cells and found that the level of SP1 protein was significantly increased in the TET1 overexpressed cells (Figures 6A,B), indicating an increase in cell viability. The above results indicated that TET1 overexpression upregulated the SP1 expression level, accelerated the gene transcription of the cells, and facilitated cell proliferation. Subsequently, we analyzed among 260 downregulated differential genes and found that DAXX was also significantly differential (Figure 4A), and WB detection of DAXX and its downstream P53 revealed that DAXX and P53 were somewhat decreased in protein level expression (Figures 5D–G), which was consistent with the previous RNA sequencing results. The above results suggest that there may be some connection between SP1 and DAXX and P53, and that DAXX co-regulates transcription and affects cell proliferation by binding to transcription factor SP1, epigenetic modifiers and chromatin remodelers.

### SP1-DAXX-TET1 Affect Self-Renewal of Spermatogonia *via* NF-Kappa B Signaling Pathway

**Figure 7:**
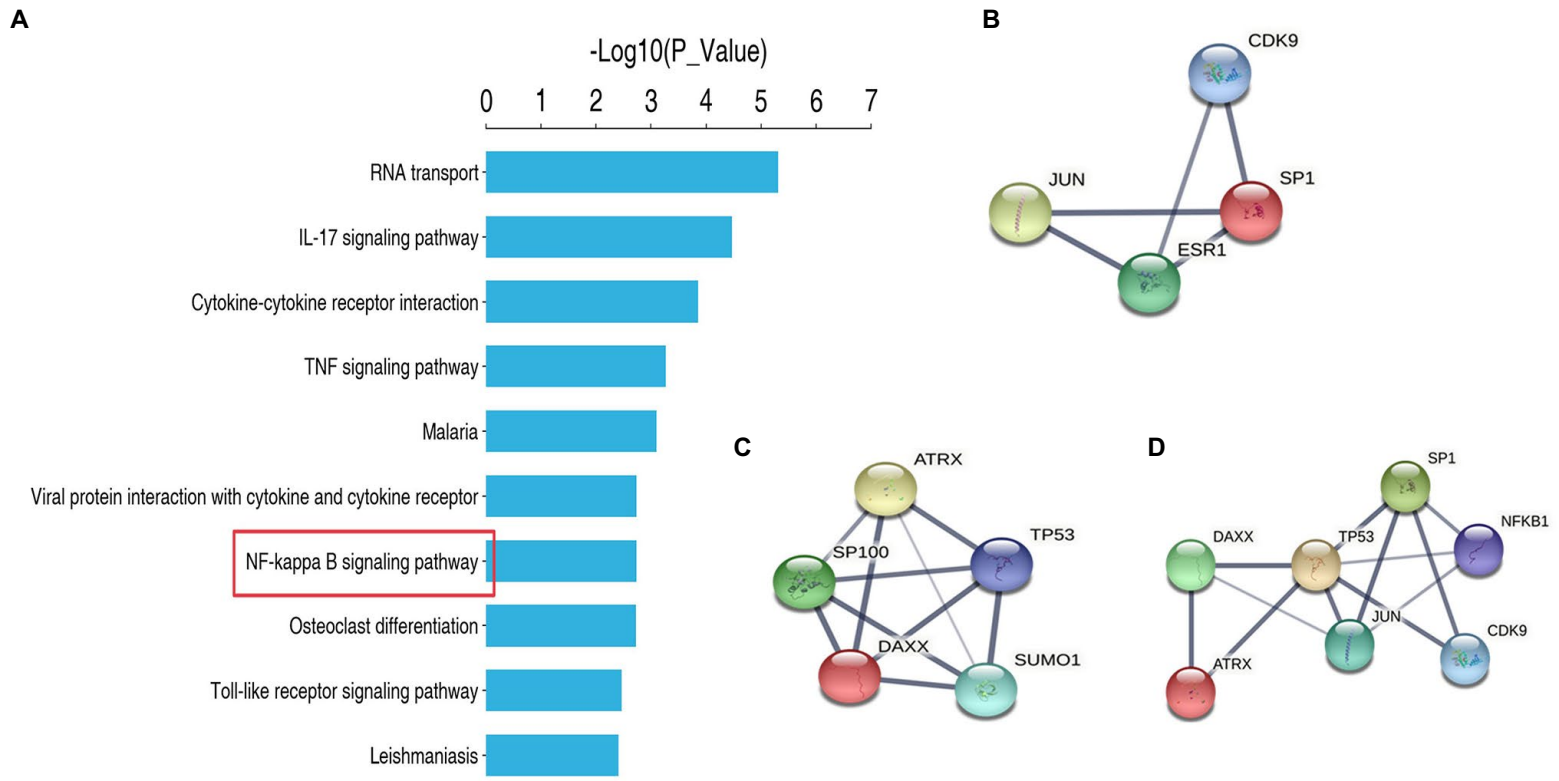
Protein sequencing analysis and protein interaction analysis. **(A)** KEGG enrichment analysis. **(B)** Protein Interaction Analysis of SP1. **(C)** Protein Interaction Analysis of DAXX. **(D)** Protein Interaction Analysis of SP1 and DAXX.

We performed protein sequencing on TET1 overexpression cells and control cells, and KEGG analysis revealed a significant enrichment of differentially expressed genes in the NF-kappa B signaling pathway, suggesting a possible co-regulation of the NF-kappa B signaling pathway (Figure 7A). Subsequently, we found multiple protein interaction patterns between significantly differentially expressed genes in the protein interaction network map (Figures 7B–D), and analysis in connection with the KEGG signaling pathway revealed that in the apoptotic signaling pathway, DAXX affects the expression of P53 by regulating downstream Jun, thus playing a pro-apoptotic role, while our transcriptome sequencing results showed that DAXX is expressed in TET1 over expression was downregulated in TET1-overexpressing cells, leading to attenuation of the pro-apoptotic effect, which may be related to the inhibition of NFKB1 that accompanies the apoptotic signaling pathway, which promotes cell survival, and transcriptome sequencing results also showed upregulation of NFKB1 expression. Secondly, we also found that SP1 was highly expressed in TET1 overexpressing cells. SP1 and its reciprocal protein CDK9 jointly regulate the transcriptional signaling pathway, and high expression of CDK9 affects its downstream KLF3, PBX3 and UTX, playing a differentiation inhibitory, pro-proliferative role. The above results suggest that there may be some antagonistic effect between SP1 and DAXX, and finally reach a dynamic balance to maintain the self-renewal of spermatogonia.

## Discussion

In this study, we determined the differential methylation sites of TET1 hydroxylation by overexpression of TET1 in spermatogonia, and combined with mRNA level and protein level analysis to further explain the regulatory mechanism of TET1 on spermatogonia self-renewal at the epigenetic level. Studies on TET1 in recent years have focused on cancer, with generally low 5hmC expression levels in patients with breast, lung, liver, gastric and pancreatic cancers, suggesting that downregulation of TET1 expression may contribute to cancer and that TET1 plays a tumor suppressive role in epigenetic modifications (Cimmino et al., 2015; Park et al., 2016; Pei et al., 2016; Collignon et al., 2018; Wu et al., 2019; Zhang et al., 2019). Moreover, TET1 can function in different cell types through different regulatory pathways (Hill et al., 2018; Damal Villivalam et al., 2020; Li et al., 2020; Smeriglio et al., 2020). Previous studies have found that TET and its intermediate 5hmC can affect male spermatogenesis through epigenetic modifications and have a crucial role in maintaining spermatogenesis (Ni et al., 2016). TET1 expression levels decline with age and TET1 is also associated with reduced fertility (Huang et al., 2020).

We examined spermatogonia-specific genes at mRNA level and protein level after TET1 overexpression and found that PLZF, a gene related to self-renewal, was upregulated at both mRNA level and protein level, indicating that TET1 has the function of maintaining spermatogonia self-renewal. The mRNA expression levels of Cylin A and Cylin E associated with cell cycle were somewhat decreased, but the expression of PCNA, a gene specific for cell proliferation, was significantly increased at the mRNA level, suggesting that TET1 overexpression did not exactly promote cell proliferation, but rather enhanced cell transcription. The mRNA expression levels of VASA, DAZL, and C-KIT, which are associated with spermatogonia differentiation, were all increased to some extent, indicating that TET1 can promote spermatogonia differentiation. All of these results verified that epigenetic modifications of TET1 play a key role in spermatogenesis and can maintain normal spermatogenesis (Zheng et al., 2016).

Subsequently, we further sequenced TET1 overexpression cells and control cells, and our analysis revealed a total of 455 differentially expressed genes, of which 195 genes were upregulated in expression at the mRNA level and 260 genes were downregulated at the mRNA level. We then used GO enrichment analysis to find that genes involved in transcriptional regulation were significantly enriched in TET1 over-expressing spermatogonia. This result suggests that TET1 overexpressing cells are more conducive to cellular transcription and thus enhanced cellular activity compared to control cells. Among these differentially upregulated genes, we found a significant upregulation of SP1, the SP family that includes SP1, SP2, SP3 and SP4, which function in various important biological processes and have been shown to have biological importance in cell growth, differentiation, apoptosis and oncogenesis (Vizcaíno et al., 2015). SP1 target genes are mainly involved in cell proliferation and tumorigenesis (Safe and Abdelrahim, 2005; Wierstra, 2008). In contrast, in previous experiments, we found that TET1 did not lead to a single proliferation due to high SP1 expression, but achieved a dynamic balance of proliferation, suggesting that SP1 may activate or repress the expression of some genes related to essential cellular functions. Our review of the literature shows the extreme complexity of SP1 function. SP1 not only activates, it also represses the expression of some essential oncogenes and tumor suppressors, and SP1 regulates some genes related to essential cellular functions, such as proliferation, differentiation, apoptosis, senescence, DNA damage response and angiogenesis. SP1 is also importantly associated with inflammation, genomic instability, and epigenetic silencing (Hanahan and Weinberg, 2000; Hanahan and Weinberg, 2011). We subsequently found significant downregulation of DAXX in differentially regulated genes. The biological function of DAXX is complex. Previous studies have identified a common denominator of interest in various cancers, namely that DAXX is overexpressed in a variety of cancers and its possible association with tumorigenesis, disease progression, and treatment resistance. DAXX can regulate transcription by binding to transcription factors, chromatin remodelers, and epigenetic modifiers. Their interactions can even directly affect apoptosis and cell signaling (Mahmud and Liao, 2019). Subsequently, we found that both SP1 and DAXX are proteins that interact with P53 through protein interaction analysis. A previous study showed that SP1 is a key factor in P53-mediated apoptosis (Li et al., 2014). Genome-wide analysis of the chromatin occupied by P53 and parallel analysis of gene expression have identified SP1 as one of the P53 regulators specific for P53-mediated transcriptional responses in the induction of apoptosis in tumor cells (Nikulenkov et al., 2012). Previous studies have identified a potential role of DAXX in the transcriptional, apoptotic and negative regulation of the P53 oncogenic pathway (Wasylishen et al., 2018). We sequenced proteins from TET1 overexpressing cells and control cells, and KEGG analysis revealed a significant enrichment of differentially expressed genes in the NF-kappa B signaling pathway and an overall upregulation of gene expression, suggesting that the NF-kappa B signaling pathway may be synergistically regulated. In the apoptotic signaling pathway, DAXX affects the expression of P53 by regulating downstream Jun, thus acting as a proapoptotic agent, while our transcriptome sequencing results showed that DAXX expression was downregulated in TET1 overexpressing cells, leading to a diminished pro-apoptotic effect, which may be related to the inhibition of NFKB1 that accompanies the apoptotic signaling pathway. KEGG analysis showed that NFKB1 could promote cell survival, and transcriptome sequencing results also showed upregulation of NFKB1 expression. Subsequently, we found that SP1 was highly expressed in TET1 overexpressing cells and that SP1 co-regulates the transcriptional signaling pathway with its counterpart, CDK9. CDK9 high expression affects its downstream KLF3, PBX3 and UTX, acting to inhibit differentiation and promote proliferation. We speculate that there may be some antagonistic effect between SP1 and DAXX, which eventually reaches a dynamic balance to maintain the self-renewal state of spermatogonia.

In summary, our results indicate that TET1 maintains self-renewal of mouse spermatogonia and facilitates cellular transcription, enhancing cellular activity, and we have identified key differential genes affected by the specific effects of TET1 and the mechanisms of interaction between these key differential genes, providing a scientific basis for studying spermatogenesis, revealing the causes of spermatogenic disorders, and elucidating their mechanisms, which may contribute to the understanding of male reproductive disorders.

## Data Availability Statement

The datasets presented in this study can be found in online repositories. The names of the repository/repositories and accession number(s) can be found at: GEO, GSE193717; ProteomeXchange, PXD030967.

## Author Contributions

LZ: conceptualization, resources, writing—review and editing, and funding acquisition. LL: methodology, formal analysis, and writing—original draft preparation. JW and SW: validation. JW: investigation. MW: data curation. YC: project administration. All authors contributed to manuscript revision, read, and approved the submitted version.

## Funding

This work was supported by the National Natural Science Foundation of China (grant no. 31902225), the Anhui Province Natural Science Fund Project (grant no. 1908085QC92), the National Natural Science Foundation of China (82173559), and the Key Research and Development Projects of Anhui Province (202104j07020035).

## Conflict of Interest

The authors declare that the research was conducted in the absence of any commercial or financial relationships that could be construed as a potential conflict of interest.

## Publisher’s Note

All claims expressed in this article are solely those of the authors and do not necessarily represent those of their affiliated organizations, or those of the publisher, the editors and the reviewers. Any product that may be evaluated in this article, or claim that may be made by its manufacturer, is not guaranteed or endorsed by the publisher.

## Footnotes

^1^ https://www.ncbi.nlm.nih.gov/geo/query/acc.cgi?acc=GSE193717
